# Generation of infectious RNA complexes in orbiviruses: RNA-RNA interactions of genomic segments

**DOI:** 10.18632/oncotarget.12496

**Published:** 2016-10-06

**Authors:** Teodoro Fajardo, Kinda AlShaikhahmed, Polly Roy

**Affiliations:** ^1^ Department of Pathogen Molecular Biology, Faculty of Infectious and Tropical Diseases, London School of Hygiene and Tropical Medicine, United Kingdom; ^2^ Current address: Nuffield Department of Medicine, University of Oxford, Oxford, United Kingdom

**Keywords:** dsRNA virus, RNA-RNA interactions, orbivirus genome assembly, segmented genome sorting and packaging, reverse genetics, Immunology and Microbiology Section, Immune response, Immunity

## Abstract

Viruses with segmented RNA genomes must package the correct number of segments for synthesis of infectious virus particles. Recent studies suggest that the members of the *Reoviridae* family with segmented double-stranded RNA genomes achieve this challenging task by forming RNA networks of segments prior to their recruitment into the assembling capsid albeit direct evidence is still lacking. Here, we investigated the capability of virus recovery by preformed complexes of ten RNA segments of H Virus (EHDV), a *Reoviridae* member, by transcribing exact T7 cDNA copies of genomic RNA segments in a single *in vitro* reaction followed by transfection of mammalian cells. The data obtained was further confirmed by RNA complexes generated from Bluetongue virus, another family member. Formation of RNA complexes was demonstrated by sucrose gradient ultracentrifugation, and RNA-RNA interactions inherent to the formation of the RNA complexes were demonstrated by electrophoretic mobility shift assay. Further, we showed that disruption of RNA complex formation inhibits virus recovery, confirming that recruitment of complete RNA networks is essential for packaging and consequently, virus recovery. This efficient reverse genetics system will allow further understanding of evolutionary relationships of *Reoviridae* members and may also contribute to development of antiviral molecules.

## INTRODUCTION

Orbiviruses (*Reoviridae* family) are vectored to particular vertebrate species (*e.g.*, sheep, cattle, horses, deer, etc.) by arthropods (gnats, ticks, or mosquitoes depending on the virus) replicating in both insect and mammalian hosts. The two most common orbiviruses are Bluetongue virus (BTV) and virus (EHDV), each with multiple serotypes. Both BTV and EHDV are transmitted from animal to animal by the same *Culicoides* vectors (gnats) and are responsible for considerable economic losses to international livestock industries [1, 2] Both viruses have been endemic in many parts of the world and recently emerged in countries and regions that were previously free of these viruses. EHDV, which is particularly endemic in the United States and Canada, causes significant in white-tailed deer (Odocoileus virginianus), however, recent reports of EHD in cattle are currently increasing, particularly in the Mediterranean basin where EHDV caused high morbidity and mortality in domestic cattle [3].

EHDV and BTV are two distinct serogroups within the orbivirus genus, but are morphologically identical with an icosahedral double-capsid structure and a genome of ten (S1-S10) double-stranded RNA (dsRNA) segments, the majority of which encode only a single protein product. The outer capsid is composed of two major proteins, VP2 and VP5, which surround the inner capsid or viral core formed by two other major proteins, VP7 and VP3 as well as the transcription complex of three minor proteins VP1, VP4 and VP6 that are closely associated with the viral genomic dsRNA segments [2, 4, 5].

Recent studies have reported that the *in vitro* packaging of the ten ssRNA segments of BTV follows a selective and sequential order, which requires specific RNA-RNA interactions and complex formation. These data implied that a preassembled network of RNA segments *in vivo* may be necessary prior to genomic RNA packaging of BTV and related Orbiviruses [6, 7]. In this study we sought to investigate if orbivirus RNA segments form a complete and pre-assembled complex of ten ssRNAs prior to genomic RNA packaging and if these RNA complexes are able to generate infectious virus particles in susceptible mammalian cells. We modified our reverse genetics (RG) system to recover infectious EHDV from RNA segments by using one of the seven EHDV serotypes, EHDV-7, as a representative, for which no RG system has been reported to date. To this end, we first generated exact cDNA copies of 10 genomic segments of EHDV-7 and assessed their suitability to generate T7 driven *in vitro* transcripts. Subsequently, ten RNA transcripts were synthesized in a single reaction to allow formation of supramolecular complexes of RNA segments and tested their infectivity in mammalian cells. The underlying RNA-RNA interactions, visualized by native gel electrophoresis, were further fractionated to separate complete RNA complexes from incomplete RNA complexes and free RNAs by sucrose gradient ultracentrifugation. Altogether, our data indicated that ssRNA segments undergo a cascade of RNA interactions required for complex formation and follow the proposed order of genome packaging for BTV, a closely related orbivirus. This RG system will also facilitate further studies on the reassortment with other serotypes or other species of the genus and to identify the regions likely to possess sorting and packaging signals.

## RESULTS

### A complete set of EHDV transcripts synthesized by a single *in vitro* transcription reaction is highly infectious: indication of formation of RNA complexes

**Figure 1:**
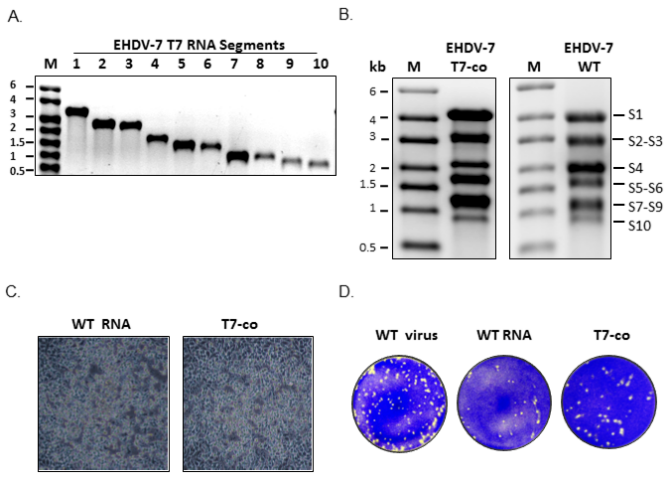
Synthesis and infectivity EHDV-7 RNA segments **A.** EHDV-7 segments S1-S10 transcribed from cDNA copies under the control of T7 promoter analyzed by 1% denaturing agarose gel. M - ssRNA standard marker **B.** Left panel, EHDV-7 RNA segments S1-S10 from simultaneous RNA synthesis of cDNA copies of S1-S10. Right panel, authentic WT RNA synthesized from viral core. Both transcription products were analyzed on a 1% denaturing agarose gel. **C.** CPE of transfected BSR cells transfected with authentic transcripts derived from viral core (WT RNA) or co-transcription products (T7-co). **D.** Plaques produced from the transfection of cells from RNA products in (B).

Emerging data on RNA assortment and packaging studies of segmented RNA viruses indicate that supramolecular RNA complexes are likely to form by specific RNA-RNA interactions, allowing selection of viral RNA segments during or prior to genome packaging and encapsidation [6-13]. To investigate further if other orbiviruses use similar strategy and to obtain direct evidence, we selected EHDV, in particular, EHDV-7, a virus for which reverse genetics (RG) system of infectious genomic RNAs are currently not available. To this end, first a set of exact cDNA copies of EHDV-7 RNA segments 1 to 10 (S1 to S10), flanked by a T7 promoter and appropriate restriction enzymes, were generated and purified as described previously [14]. Ten transcripts were synthesized using these cDNA templates and 1% denaturing MOPS agarose gel analysis demonstrated that each transcript had the correct molecular size (Figure 1A). Subsequently, approximately equimolar amounts of S1 to S10 cDNA template were mixed and RNA transcripts were synthesized simultaneously in a single reaction (co-synthesis). The *in vitro* co-transcription products of the EHDV-7 segments showed that ssRNA bands were synthesized in correct sizes similar to the authentic transcripts derived from wild type (WT) viral core (Figure 1B). However, due to small differences in their molecular sizes, certain RNA bands migrated closely (S2-S3, S5-S6, S7-S9) in this denaturing agarose gel and were difficult to distinguish as discrete bands. To evaluate if RNA products of the co-transcription and authentic viral transcripts (WT RNA derived from purified cores *in vitro*) are infectious, confluent BSR monolayers were transfected first with S1, S4, and S9 (coding for primary replicase proteins VP1, VP4 and VP6) along with S3 (VP3), S5 (NS1) and S8 (NS2) as reported previously [15, 16] and incubated at 35°C for 18h. The monolayer cells were then transfected with the complete set of co-transcribed 10ssRNA or WT RNA transcripts. At 72 hours post-transfection (hpt), a cytopathic effect indistinguishable from that caused by WT EHDV-7 virus infection, was detected in ~80% of cells transfected by both RNA products (Figure 1C), demonstrating that infectious virus particles were produced. Subsequently, to validate virus recovery by both reaction products, BSR monolayers were transfected with ~1μg of T7 RNA transcripts, WT RNA or wild-type virus and overlaid with agarose. Plaques in the transfected cells of both RNA products were visually similar in appearance to those produced by infection with WT EHDV-7 virus (Figure 1D). However, the T7 derived co-transcript mixture appeared to generate more virus plaques than the authentic viral transcripts at an approximately same concentration of RNA, suggesting that the virus recovery from *in vitro* co-synthesized transcript mixture is highly efficient.

**Figure 2:**
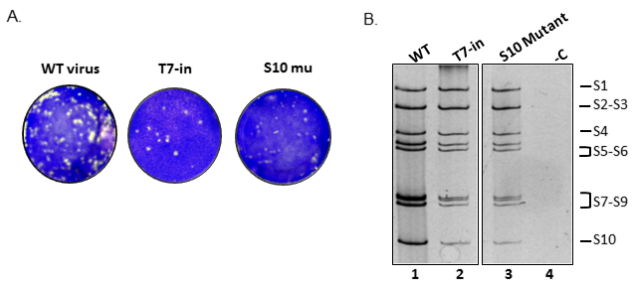
Reverse genetics of individually synthesized segments **A.** Plaques produced from the transfection of cells from individual EHDV-7 RNA segments S1-S10 (T7-in), WT or S10 mutant (S10 mu). **B.** dsRNA profile of EHDV-7 virus on a 10% PAGE recovered by RG using individually synthesized S1-S10 RNA segments (WT or mutant S10), (−C) non-infected cells.

Although the above data demonstrates that the mixture of co-transcribed RNA segments is infectious, it was necessary to prove that a mixture of individually synthesized and purified segments is also infectious in BSR cells as reported for other orbiviruses [14, 15, 17-20]. Therefore, a set of cDNA copies of each EHDV-7 RNA segments S1 to S10 were generated and purified as described previously [14] and ~100 ng each of the ten RNA segments were transfected onto BSR cells as described in Materials and Methods. Viral plaques generated by the purified individual transcripts (Figure 2A, T7-in) appeared at 72 hpt, indicating a successful infection with comparable plaque size to that of the WT virus. The genomic dsRNA profile from an amplified plaque also showed a typical EHDV-7 genomic dsRNA profile (Figure 2B, T7-in).

To ensure that the rescued virus was not from contaminating WT EHDV-7, a silent mutation in the S10 open reading frame (ORF) was introduced to abolish the *NdeI* restriction site (T to A mutation at position nt416) and a mutant virus was successfully recovered with the S10 mutant together with the S1-S9 wild type transcripts. No changes could be detected in the dsRNA profile of the mutant S10 when compared with that of the WT virus (Figure 2B). RT-PCR of the purified S10 mutant virus demonstrated the absence of the *NdeI* restriction site, a silent mutation in the S10 open reading frame (ORF), which was further confirmed by sequencing (data not shown). The successful recovery of virus particles from transfection of individual RNA segments confirmed the ability of the RNA transcripts to generate viable virus, albeit with a lower number of virus plaques compared with the co-synthesized transcripts at an approximately equal amount of transfected RNA segments.

**Figure 3:**
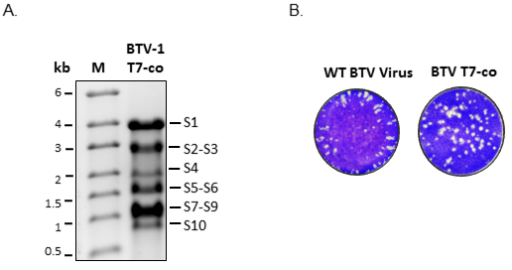
Synthesis and infectivity BTV-1 RNA segments **A.** Co-transcription products of BTV-1 segments S1-S10 cDNA copies under the control of T7 promoter analyzed on a 1% denaturing agarose gel. M - ssRNA standard marker **B.** Plaques formed in cell transfected with WT BTV-1 or T7 Co-transcription products.

To substantiate the efficient recovery of EHDV-7 virus from transfection of the co-synthesized transcripts, we also utilized the same approach for recovery of BTV, another orbivirus. Using previously synthesized T7 cDNA of ten BTV-1 segments [14], all 10 ssRNAs were co-transcribed in a single transcription reaction as described. As shown in Figure 3A, the BTV RNA transcripts were synthesized at the correct sizes and transfection of mammalian cells using the *in-vitro* co-synthesized RNA transcripts yielded viral plaques that started to appear at 48hpt, and 100% CPE at 96hpt (Figure 3B), similar to the results observed using EHDV-7 co-synthesized transcripts. These results indicated that the efficient infectivity of co-synthesized transcripts was possibly due to the interactions of all segments forming a single RNA complex, necessary for recruitment, and likely shared by other orbiviruses and related segmented dsRNA viruses.

### Co-synthesis of ten ssRNA transcripts triggers RNA complex formation

**Figure 4:**
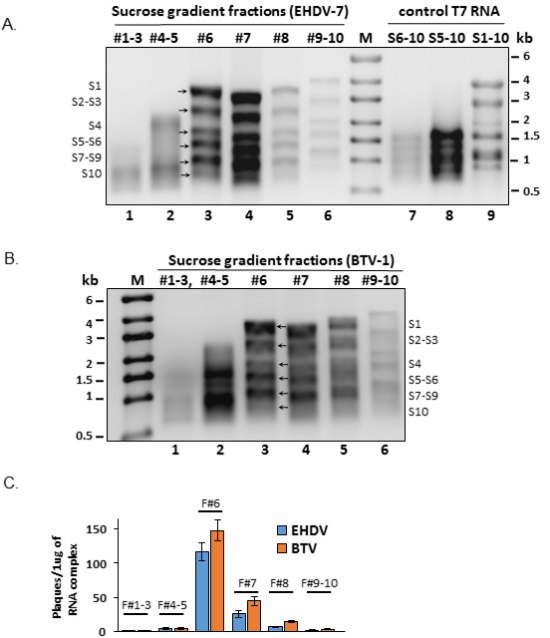
Formation and fractionation of S1-S10 RNA complexes **A.** Co-transcription products of EHDV-7 S1-S10 segments fractionated on a 15-65% sucrose gradient and analyzed on a 1% denaturing agarose gel. Co-transcripts S6-S10, S5-S10 and S1-S10 from T7 cDNA were also analyzed as marker control. **B.** Co-transcription products of BTV-1 S1-S10 segments fractionated on a 15-65% sucrose gradient and analyzed on a 1% denaturing agarose gel. **C.** Virus plaque yield from ~1μg RNA complex fractionated by sucrose gradient ultracentrifugation and transfected on BSR cells, *n* = 3.

Efficient recovery of infectious EHDV-7 virus from the transfection of co-synthesized 10 ssRNA products suggests that the 10 transcripts may organize into an RNA complex that is infectious. To investigate this possibility, a single transcription reaction was undertaken using T7 cDNA templates of all 10 segments as described above, and the RNA product was analyzed by ultracentrifugation on a 15-65% sucrose gradient followed by fractionation from the top. When the RNA contents of each fraction were analyzed by denaturing gel electrophoresis the majority of gradient fractions had ssRNA molecules. The ssRNAs corresponding to the correct sizes of all 10 ssRNA segments (complete RNA complex of 10 segments) were detectable mainly in two fractions at the middle of the gradient, fractions #6 and #7 (Figure 4A). Complexes of distinct size, smaller or incomplete (top fractions #1-3 and #4-5) were also detected (Figure 4A). These smaller complexes moved to the size corresponding to small and medium size RNA segments when compared to the molecular marker and the control S1-S10 RNA segments, suggesting that these were likely to be the free RNA molecules not associated with the complete complex. To substantiate the results of EHDV-7 RNA complex formation, we performed the same experiment using BTV-1 as an alternate orbivirus species (Figure 4B). The results that we obtained after fractionating and denaturing agarose gel analysis of the co-transcribed S1-S10 segments were identical with that of the EHDV-7 RNA segments, indicating that the 10 genomic RNA of these two orbiviruses can organize into single high molecular mass RNA complex when all of the segments are simultaneously transcribed.

The infectivity of the fractionated RNA complexes was also tested by transfection of BSR cells as described. Typical viral plaques were recovered from fractions #6 and #7 while few or no virus plaques developed from the rest of the fractions (Figure 4C). These results revealed that only fractions with a complete set of 10 ssRNA in fractions #6 & #7 were able to generate viable viruses, suggesting that the pre-formed RNA complexes migrating in these fractions were the complete set of infectious 10 ssRNA network. Infectivity of the fractionated complexes was also reproducible for BTV-1, and similar results were obtained as shown in Figure 4C. The results of the infectivity assay of fractionated co-transcription products indicated that the ssRNAs are interacting with each other and forming RNA complexes in stages starting from the interactions of the smaller sizes of RNAs to a complete network of 10ssRNAs. This data is consistent with our previous findings that the BTV RNAs are organized and packaged in a sequential manner according to segment size classes [6, 7]. The data also indicated that nascent ssRNAs synthesized together at the same time were more highly likely to interact with other segments to form an infectious RNA complex of 10 RNA segments.

### Specific RNA-RNA interactions are essential for the infectivity of RNA complexes

**Figure 5:**
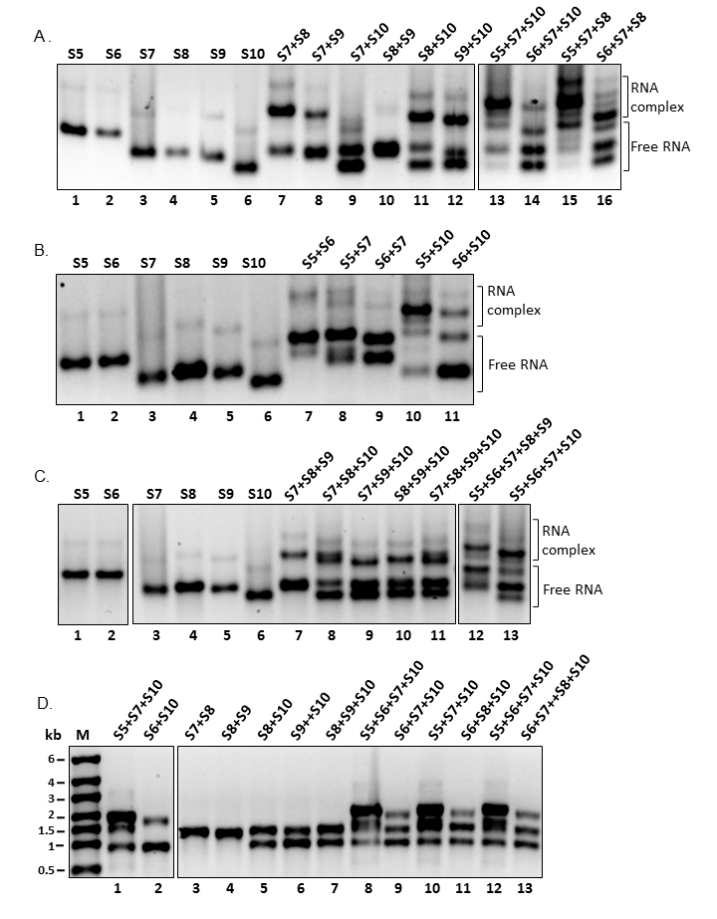
Interactions and complex formation of RNA segments showing the complex and free RNAs **A.** Interactions of two and three co-transcribed small EHDV-7 RNA segments **B.** RNA-RNA interactions of medium sized and S5 or S6 + S10 EHDV-7 segments **C.** Interactions of multiple RNA combinations from co-transcription of small EHDV-7 RNA segments (RNA interactions of **A.**-**C.** were analyzed on a 1% native agarose gel electrophoresis in TBM buffer as described. **D.** Selected reactions from (A), (B) and (C) showing the integrity and mobility on a 1% denaturing gel electrophoresis in MOPS buffer.

The sucrose gradient data indicated that the ssRNA complexes are formed in stages. Further our previous data also demonstrated that BTV ssRNA segments interact with each other in a highly specific manner which is probably necessary for forming a network of ssRNA molecules prior to packaging [7]. To determine whether RNA-RNA interactions occur and could be identified between different EHDV RNA segments, T7 transcripts in combinations of two, three or multiple medium and small segments (from S5-S10) were co-synthesized in single transcription reactions. The co-transcribed reaction products were subsequently analyzed for RNA-RNA interactions and RNA complex formation by native agarose gel electrophoretic mobility shift assay (EMSA). Differences in the degree of RNA-RNA interactions as indicated by the bound (retarded) RNA forming complexes were observed between and among RNA segments. Of the two RNA segment combinations, the strong interactions were detected between the four small RNA segments (S7+S8, S7+S9, S8+S10 and S9+S10), except the combination of S8 and S9, both of which interacted with S10 or S7 (Figure 5A, lanes 7-12). In contrast to the smaller RNA segments, interaction between medium size segments, S5 and S6 with S7 was particularly weak (Figure 5B). However, the weak interactions were noticeably enhanced when the interacting RNA partner was replaced by S10 (S5+S10, S6+S10), (Figure 5B, lanes 10 and 11) indicating the binding preference of these segments to the smallest segment S10, which also suggest that S10 has a crucial role in complex formation of the RNA segments, consistent with our previous hypothesis of its leading role in sorting and packaging of BTV RNA segments [7].

In the combinations of three RNA segment interaction assays, distinct RNA complexes were detected when S5 or S6 was included in the reaction with segments S7, S8 and S10 (Figure 5A, lanes 13, 14, 15 & 16) suggesting that both S5 and S6 formed stronger complexes only when smaller segments were present in the interactions. In contrast to the requirement of the medium segment S5 and S6 to form stable RNA interactions, the four smaller segments, S7, S8, S9 and S10 formed strong complexes in three segment combinations (S7+S8+S9, S7+S8+S10, S7+S9+S10 and S8+S9+S10) indicating that RNA-RNA interactions readily occurred between smaller EHDV RNA segments (Figure 5C, lanes 7-11).

As expected, strong RNA complexes were detected when four or five RNA segments (S5-S10) were mixed in a single transcription reaction (Figure 5C, lanes 11-13) suggesting that non-interacting segments could only form RNA complexes when partner RNA segments were present in the reaction mixture, probably due to base pairing to their binding sequences. Also, the formed RNA complex bands in the two or multiple RNA combinations were also markedly distinct and stronger than the very faint homodimers from the single RNAs. The formation of strong RNA complexes between and among medium and small segments synthesized in a single reaction indicate that nascent RNA segments readily recruit other RNA segments and assemble into complexes, only when their interacting partner RNA segments are present. These data also suggest the RNA-RNA interactions are highly specific between segments. The synthesis of ssRNAs in the reaction products were also analyzed by denaturing MOPS gel, which demonstrated that the transcripts of all segments were synthesized in correct sizes in the presence of other segments (Figure 5D).

### Disruption of the pre-formed 10ssRNA complexes reduces the efficiency of virus recovery

**Figure 6:**
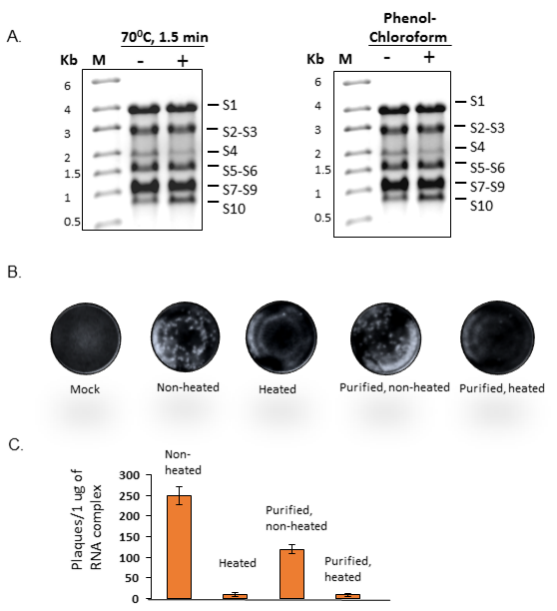
Infectivity of disrupted co-transcribed RNA products **A.** Co-transcribed product of EHDV-7 S1-S10 were treated at 70°C or phenol-chloroform purified and analyzed on a 1% denaturing agarose gel in MOPS buffer. **B.** Viral plaques from transfection of cells using the RNA products from (A). **C.** Histogram of the quantified plaques developed after transfection with the fractionated RNA complex, *n* = 3.

The results obtained from the above experiments demonstrated that RNA complexes were formed *via* RNA-RNA interactions and that the infectious complexes were sufficiently stable to tolerate isolation by sucrose gradient centrifugation. It was therefore imperative to confirm that disruption of RNA complexes would affect the virus recovery. To validate this, the 10 ssRNA transcripts were co-synthesized and products were divided into two aliquots. While one aliquot was subjected to heat treatment followed by flash cooling as described in Materials and Methods, the other was kept as control. BSR cells were then transfected with both aliquots and incubated for plaque formation. Results showed that the heated RNA products generated a markedly reduced number of plaques when compared to the untreated control (Figure 6A, left panel). In parallel, a second co-transcription reaction was also undertaken and an aliquot of the RNA complex products was then purified by standard phenol-chloroform extraction followed by lithium chloride precipitation, and then subjected to heat treatment as described (Figure 6A, right panel). A similar reduction in virus recovery was observed with treated product when compared to the non-treated control (Figure 6B and 6C). The denaturing agarose gel of the heated and purified RNA complexes in Figure 6B showed that the 10 ssRNAs were still complete even after purification and heating process prior to transfection as compared to the non-treated complex analyzed on the same denaturing gel. These data suggest that reduction of the virus recovery after treating the complex with heat or purification with phenol chloroform have possibly affected the inter-segment interactions, essential for RNA complex formation.

## DISCUSSION

Recent developments on intersegment interactions for dsRNA viruses suggest that segments form a RNA complex through RNA-RNA interactions for genome organization [6, 7, 9-11, 21]. Interactions among segments are also thought to be the mechanisms involved for the generation of viable virus by RG, which is principally focused on transfection of 10 authentic ssRNA transcripts synthesized from purified viral core [17, 22] or individual ssRNAs synthesized from T7 cDNA plasmid clones followed by pre-incubation and transfection [14, 18], [23]. Here, we showed that a mixture of ten individually transcribed EHDV-7 RNA could effectively recover progeny virus particles. Moreover, we also demonstrated that it was possible to bypass this cumbersome system by devising a novel method in which all T7 transcripts were synthesized simultaneously in a single reaction and RNA complex generated viable viruses in transfected cells more efficiently. This system has also allowed us to further our understanding of the mechanism of orbivirus replication, where genome segments are required to interact with each other for formation of stable complexes prior to being packaged and encapsidated [6, 7, 11].

It was also shown for the first time that infectious EHDV or BTV was recovered from pre-formed RNA complexes (co-synthesized RNA) upon transfection of cells. Virus recovery was achieved not only at a higher number but were also detected earlier than that of the individually synthesized ssRNAs. These results highlighted that the key factor for the successful recovery of infectious viruses was due to ssRNA complexes allowing specific RNA-RNA interactions prior to transfection as suggested previously [6, 7, 24]. This modified RG system also parallels the natural situation of orbivirus infection (EHDV and BTV), where simultaneous transcription of all RNA segments within the transcriptionally active viral cores (where replicase or transcriptase complex resides) and their subsequent release in to the host cell cytoplasm occurs at the same time. Whether genome packaging is accomplished by chance or randomly incorporating all genomic segments or through a selective process is still not fully understood. The rapid virus recovery therefore, is probably due to the replicase complex readily recognizing the incoming preformed 10 ssRNA network, and there was no lag period necessary for sorting and packaging process.

Intersegment interaction of all BTV segments is important in orbivirus genome sorting and packaging as earlier proposed in the cell-free assembly assay system [24] and confirmed further by demonstrating intersegment interactions and sequential packaging order [6, 7, 11]. The disruption of the essential RNA interactions between segments in the pre-formed RNA complexes by heat treatment or phenol-chloroform purification followed by heat treatment further showed reduced virus recovery in transfected mammalian cells compared to the untreated RNA complexes. This underscores the possibility of a stable intersegment RNA interactions as pre-requisite for the infectious RNA complex formation.

Separation of 10 ssRNA complexes of EHDV-7 and BTV-1 from the incomplete RNA complexes provided further evidence that higher order RNA complexes are formed through specific RNA-RNA interactions. Such events must occur prior to RNA complex assembly. The isolation of smaller complexes corresponding to small and medium size class of RNA segments has also supported our previous findings that BTV RNA complex are formed through a coordinated interactions of RNA starting from forming the smaller complex of smaller segments and recruits the next size classes [6, 7].

Using a unique *in vitro* assembly of the BTV capsid, it was demonstrated that ssRNA copies of all 10 genomic segments were needed to generate an infectious viral core and that in absence of certain segments ssRNAs were not packaged [7, 24]. It was hypothesized that during genome assembly, nascent ssRNAs most likely interacted with each other through RNA-RNA interactions, thereby sorting the correct set and number of RNA segments for encapsidation. Moreover, studies carried out on the dsRNA synthesis of single-shelled insect cytoplasmic polyhedrosis virus (CPV) has shown that the ten segmented dsRNAs in CPV are organized with ten transcriptional enzyme complex of RNA-dependent RNA polymerase (RdRP) and NTPase VP4 (TEC) in a specific, non-symmetric manner, with each dsRNA segment attached directly to a TEC while in while in cypoviruses (NCPV and TCPV) each RdRP is anchored at the inner surface of the capsid and surrounded by multiple layers of dsRNA [25, 26]. In this study, we have shown evidences of intersegment interactions between medium and small size class initiation of stable RNA complexes. This is consistent with our previous findings [6, 7] and similar studies by others on other segmented viruses where some segments appeared to have preferential binding affinity to enhance formation of the RNA network [8, 27, 28].

The successful recovery of viable virus using approaches we introduced here has proven the likelihood of the hypothesis that RNA segments make RNA contacts for correct genome organization, and suggests that a similar pathway linked to RNA-RNA interactions and complex formation occurs between related orbiviruses.

Understanding how orbivirus organizes its multiple genetic material into one compact complex of RNA ready for another round of virus life cycle either for encapsidation or gene expression is fundamental for an effective control of this economically important virus. Broader studies on RNA-RNA interactions of segmented viruses can also be used to effectively inhibit network formation through designer oligonucleotides, siRNA or virus delivery system and thereby virus multiplication. This study has also established an improved and facile RG system for *Reoviridae* and opened up an opportunity to investigate evolutionary relationship between different members of orbivirus species.

## MATERIALS AND METHODS

### Cells and virus

BSR cells, a BHK 21 clone derivative of baby hamster kidney cells (American Type Culture Collection) were grown in Dulbecco modified Eagle medium supplemented with 5% fetal calf serum (FCS), penicillin, streptomycin and amphotericin B at 35°C with 5% CO_2_.

Bluetongue virus serotype 1 (BTV-1) South African reference strain and Epizootic hemorrhagic disease virus serotype 7 (EHDV-7) (CSIRO 775 strain) were plaque purified and amplified in BSR cell line. Virus stocks were maintained by infecting BSR cells at multiplicity of infection (MOI) of 0.1 and harvested at 100% cytopathic effect (CPE).

### Synthesis of EHDV cDNA, individual transcripts and mutagenesis

Full-length cDNA copies of each genomic segment of EHDV-7 flanked by T7 promoter and restriction enzymes were synthesized using the full-length amplification of cDNAs (FLAC) methodas described [14] with some modifications. Briefly, genomic dsRNAs were isolated from infected cells and cDNA was generated from each segment and amplified using FLAC. Each segment was then cloned and sequenced. A mutated construct of EHDV S10 with deletion of *Nde*I restriction site was generated by overlapping polymerase chain reaction (PCR) through site-directed mutagenesis and treated with *Dpn*I to digest the parental plasmid [29]. Complete set of ten EHDV-7 (S1-S10) capped T7 transcripts were synthesized The capped T7 transcripts were synthesized using mMESSAGE mMACHINE T7 Ultra Kit (Ambion) and analyzed on a 1% denaturing agarose gel in MOPS (morpholinepropanesulfonic acid) buffer with formaldehyde followed by ethidium bromide staining. The synthesized transcripts were resuspended in nuclease-free water and stored at −80 C until used for virus recovery using reverse genetics system as described [14].

### Generation of 10ssRNA from co-transcription of cDNA copies or from virus core for reverse genetics

For synthesis of EHDV and BTV co-transcripts, exact cDNA copies of EHDV-7 or BTV-1 genome segments S1-S10 were used as templates in one single transcription reaction using mMESSAGE mMACHINE T7 Ultra Kit (Ambion) according to manufacturer's procedures. For generation of transcripts expressing the proteins necessary for primary replication, S1, S3, S4, S6, S8 and S9 plasmids were co-transcribed to facilitate the synthesis of VP1, VP3, VP4, VP6, NS1 and NS2, respectively in the transfected cells. For synthesis of RNA transcripts from viral core, EHDV cores were purified according to the modified methods described previously [30, 31]. Briefly, EHDV core particles were incubated at 40 μg/ml at 30°C for 5 to 6 h in core transcription buffer (100 mM Tris-HCl, pH 8.0, 4 mM ATP, 2 mM GTP, 2 mM CTP, 2 mM UTP, 500 μM*S*-adenosylmethionine, 6 mM dithiothreitol, 9 mM MgCl_2_, 0.5U/μl RNasin Plus (Promega) [22]. Both transcription products were used for the recovery of the virus using the modified reverse genetics system described below. The integrity of all transcribed RNAs were routinely checked on a 1% denaturing agarose gel electrophoresis in MOPS buffer and formaldehyde.

### Modified reverse genetics system

For the reverse genetics system, ~1 μg of the co-synthesized RNA products (S1, S3, S4, S6, S8 and S9) were transfected directly onto a 90% confluent cells (1.5×10^5^) and incubated for 16-18 h followed by a second round of transfection with ~1.5 μg of 10ssRNA co-transcripts or authentic viral transcripts and overlaid with 0.8% agarose gel in DMEM with 1% FCS. The cells were further incubated for up to 72 h and observed for virus plaque formation. To confirm virus recovery, plaques from the reverse genetics were picked and amplified and genomic dsRNAs were extracted, and analysed on a 10% native PAGE

### RNA complex formation, separation by ultracentrifugation and transfection

For RNA complex formation,150 ng linearized plasmid of each segments were co-transcribed (S1-S10) in a buffer containing 40mM Tris, pH7.5; 10mM MgCl_2_; 20mM NaCl_2_; 3mM spermidine, 50mM DTT; 5mM each rNTPs; 10U RNase inhibitor and 40U of T7 RNA polymerase (Thermo Scientific) for 3 h at 37°C followed by RNase free DNase 1 treatment. Immediately after, the reaction mixture was loaded on a 15% to 65% continuous sucrose gradient for 90 min at 200,000 ×*g*at 4 °C, using a TLS55 rotor. Fractions were collected from the top and used for the second transfection of BSR cells pre-transfected with (S1, S3, S4, S6, S8 and S9) transcripts for recovery of viable virus. The fractionated complexes were analyzed on a 1% denaturing agarose gel electrophoresis in MOPS buffer and formaldehyde to check the integrity of transcription.

### RNA-RNA interaction and electrophoretic mobility shift assay (EMSA)

For RNA-RNA interactions of EHDV RNA segments, 150 ng linearized plasmid of each segments (S5-S10) were transcribed either in pairs or combinations of multiple segments. RNA transcription was carried out in a buffer containing 40mM Tris, pH7.5; 10mM MgCl_2_; 20mM NaCl_2_, 3mM spermidine, 50mM DTT; 5mM each rNTPs; 10U RNase inhibitor and 40U of T7 RNA polymerase (Thermo Scientific) for 3 h at 37°C followed by RNase free DNase 1 treatment. Immediately after DNase treatment, detection of RNA complexes was done by electrophoretic mobility shift assay (EMSA). Electrophoresis gel was run for 180 min at 150 V in TBM buffer (45mM Tris, pH8.3; 43mM boric acid; 0.1mM MgCl_2_) and stained with 0.01% (w/v) ethidium bromide. The integrity of transcribed RNA was checked routinely by denaturing gel electrophoresis.

### Disruption of RNA-RNA interaction by heat or phenol-chloroform treatment

EHDV RNA segments were allowed to form a complex by co-transcription of S1-S10 as described. Immediately after DNase treatment, an aliquot was collected and heat treated at 70°C for 1.5 minutes and flash cooled on ice. Another aliquot was treated by standard phenol chloroform extraction and purified RNAs were suspended in RNase free water before heating at 70°C for 1.5 minutes and flash cooled. Both aliquots were used for the second transfection of BSR for recovery of viable virus. The integrity of transcribed RNA was checked by denaturing gel electrophoresis.
